# Allocation of Eavesdropping Attacks for Multi-System Remote State Estimation

**DOI:** 10.3390/s24030850

**Published:** 2024-01-28

**Authors:** Xiaoyan Chang, Lianghong Peng, Suzhen Zhang

**Affiliations:** Shandong Key Laboratory of Industrial Control Technology, School of Automation, Qingdao University, Qingdao 266071, China; cxy991028hhhhh@163.com (X.C.); zsz19990316@163.com (S.Z.)

**Keywords:** cyber-physical systems (CPS), kalman filtering, eavesdropper, markov decision processes (MDP), optimization algorithm

## Abstract

In recent years, the problem of cyber–physical systems’ remote state estimations under eavesdropping attacks have been a source of concern. Aiming at the existence of eavesdroppers in multi-system CPSs, the optimal attack energy allocation problem based on a SINR (signal-to-noise ratio) remote state estimation is studied. Assume that there are *N* sensors, and these sensors use a shared wireless communication channel to send their state measurements to the remote estimator. Due to the limited power, eavesdroppers can only attack *M* channels out of *N* channels at most. Our goal is to use the Markov decision processes (MDP) method to maximize the eavesdropper’s state estimation error, so as to determine the eavesdropper’s optimal attack allocation. We propose a backward induction algorithm which uses MDP to obtain the optimal attack energy allocation strategy. Compared with the traditional induction algorithm, this algorithm has lower computational cost. Finally, the numerical simulation results verify the correctness of the theoretical analysis.

## 1. Introduction

Cyber–physical systems (CPS) are considered to be among the revolutionary technologies due to the continuous technological breakthroughs and innovations in information technology and in the manufacturing industry [1]. CPS is a multidimensional and complex system that deeply integrates control, communication and computing (that is, 3C technology composed of control, communication and computing)and can realizes large-scale information acquisition and intelligent control of the physical world through the cognition, communication and control of physical objects, so that the network can monitor the specific actions of a physical entity in a real-time, reliable, remote and safe way [2,3]. CPS is widely used in aerospace, industrial production, advanced automobile systems, energy reserve, environmental monitoring, national defense and military, infrastructure construction, intelligent building, smart grids, transportation systems and telemedicine [4]. With the rapid development of network, computing, sensing and control systems, CPS technology is more and more widely used, and the emerging network attacks make the wireless CPS system very fragile, and the security of CPS becomes the primary consideration [5,6,7].

For the security issues of a system’s remote state estimation, there are many forms of malicious network attacks, but they are divided into three main and common categories: denial of service (DoS) attacks, integrity (including replay and false data injection) attacks and eavesdropping attacks [8]. DoS attacks are designed to interfere with wireless communication channels. This attack will lead to a significant decline in the estimation accuracy in CPS [9]. Peng [10] and Zhang [11] formulated the problem as a Markov decision process (MDP) problem to consider the optimal attack power allocation for remote state estimation in a multi-system. Integrity attacks can disrupt the transmitted data packets with stealth constraint [12,13]. In Ref. [14], an important scenario is designed from the attacker’s point of view, in which the false data injection attack can completely and secretly destroy CPS. In addition, the channel may be subject to eavesdropping attacks, which can lead to serious economic losses and even pose a threat to human survival by eavesdropping on personal privacy data [15,16]. For example, in the intelligent transportation system, eavesdroppers infer the path planning of vehicles by monitoring the location information, and on this basis, eavesdropping attacks will easily succeed [17,18]. In terms of existing research, data encryption is the main method to protect system privacy from eavesdropping attacks [19,20,21].

Recently, the issue of remote state estimation in the presence of eavesdroppers has attracted widespread attention from researchers. The attack types of eavesdropping attacks are divided into passive eavesdropping attacks and active eavesdropping attacks. Some estimation and control problems have been studied in the presence of active attacks. Han [22] studied the problem of active eavesdropping on fading channels, and proposed an interference-assisted eavesdropping method to improve the probability of successful monitoring. Yuan [23] constructed a two-person non-zero-sum game between the sensor and the active eavesdropper with the goal of minimizing the covariance of the self-estimated error and maximizing the covariance of the opponent’s estimated error. Ding [24] took the trade-off between stealth and eavesdropping performance as a constrained MDP, and proposed an optimal strategy for active eavesdropping.

The above literatures indicate a certain breakthrough in the design of active eavesdropping solutions. This paper mainly studies the passive attacks of eavesdroppers. Tsiamis [25] proposed a confidentiality mechanism for randomly hiding sensor information, and explored the trade-off between user utility and control theory confidentiality through optimization methods. Huang [26] proposed a new encryption strategy and considered the cost of the encryption process. Then, the optimal determinism of the encryption strategy and the existence of the Markov strategy in the finite time horizon are proven. Wang [27] theoretically proved that there are some structural properties in the optimal transmission scheduling for known and unknown eavesdropper estimation errors. In reference [28], the transmission scheduling strategy of remote state estimation systems with eavesdroppers on packet-dropping links was studied. Yuan [29] transformed the system model into MDP in order to obtain the optimal transmission scheduling to minimize the AoI of CPS and keep the AoI of eavesdroppers above a certain level, and proved that the optimal transmission scheduling strategy is a threshold behavior on the CPS and AoI of eavesdroppers, respectively. In [30], the proposed problem is formulated as a Stackelberg game, and the strategy of maximizing the secure transmission rate between sensor and controller in the presence of malicious eavesdroppers and disruptors is studied. On the basis of analyzing the influence of different strategies on eavesdropping performance, Zhou [31] studied the multi-output system and proposed a decryption scheduling scheme to minimize the expected estimation error under the condition of energy constraint.

Most of the existing literature studies the optimal transmission strategies of sensors from the remote estimator. Compared to [27,28], this paper studies the optimal attack energy allocation strategies from the perspective of eavesdroppers. Moreover, the previous literature mainly focuses on the situation that CPS has eavesdroppers in a single system and a finite time range, but does not pay too much attention to the situation when there are eavesdroppers in a multi-system and in an infinite time range. In this paper, the optimal attack allocation problem of remote state estimation in CPS with eavesdropping attacks in a multi-system in infinite time range is studied. Our goal is to maximize the state estimation error of the eavesdropper, so as to determine the optimal attack allocation of the eavesdropper. The contributions of this paper are as follows:

1. We propose a multi-system eavesdropping attack model based on channel SINR, which reveals the relationship between attack power and packet arrival rate.
2. In the infinite time horizon, under the condition of energy constraint, the optimal attack scheduling strategy is obtained by constructing MDP and using the Bellman equation.
3. Finally, according to the given algorithm, the optimal attack energy allocation strategy is obtained, and then it is verified by simulation experiments.

Notations: The entire paper uses the following symbols. N is the set of natural numbers. The *n*-dimensional Euclidean space is denoted by $R^{n}$. $S_{+}^{n}$ ($S_{++}^{n}$) is the set of *n* by *n* positive semi-definite matricess (and positive definite matrices). Tr(X) is the trace of a matrix *X*, and $X^{T}$ is the transpose of *X* and $X^{-1}$ denotes the inverse of matrix *X*. X>0 and X≥0 represent that *X* is a positive definite matrix and positive semidefinite matrix, respectively. For functions $\tilde{g}\;$ and *h*, $\tilde{g}\circ h\left(x\right)$ stands for the function composition $\tilde{g}\left(h\left(x\right)\right)$ and $h^{n}\left(x\right)=h\left(h^{n-1}\left(x\right)\right)$ with $h^{0}\left(x\right)=x$. E[·] indicates taking the expected value of ′·′. P[·] denotes the probability of ′·′.

## 2. Problem Setup

### 2.1. System Model

**Figure 1:**
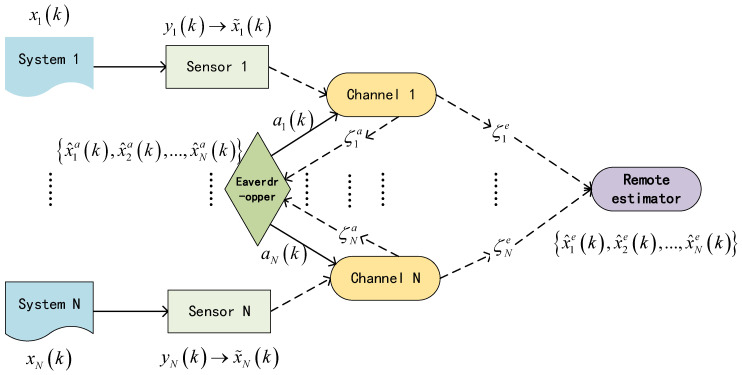
System architecture.

Figure 1 shows the system architecture. We consider *N* general discrete time-invariant stochastic system, which is given as follows

(1) $$\begin{array}{ccc} x_{i}\left(k+1\right) & = & A_{i}x_{i}\left(k\right)+\omega_{i}\left(k\right), \end{array}$$

(2) $$\begin{array}{ccc} y_{i}\left(k\right) & = & C_{i}x_{i}\left(k\right)+v_{i}\left(k\right) \end{array}$$

where k∈N is the time index and $x_{i}\left(k\right)\in R^{n}$ and $y_{i}\left(k\right)\in R^{m}$ refer to the state of the *i*th system and the system measurements vector taken by the sensor at time *k*, respectively. The process noise $\omega_{i}\left(k\right)\in R^{n}$ and the observation noise $v_{i}\left(k\right)\in R^{m}$ are assumed to be independent and identically distributed (i.i.d.) Gaussian noises with zero-mean and the covariances matrix $Q_{i}⩾0$ and matrix $R_{i}>0$, respectively. The initial state of the *i*th system $x_{i}\left(0\right)$ is also a zero-mean Gaussian random variable independented $\omega_{i}\left(k\right)$ and $v_{i}\left(k\right)$ with covariance $F_{i}\left(0\right)\geq 0$. We also assume that the pair $\left(A_{i},C_{i}\right)$ is observable and $\left(A_{i},\sqrt{Q_{i}}\right)$ is stabilizable. Assuming that the sensors in the system are intelligent sensors with certain computing powers, each sensor can first use the collected observation data to calculate the local state estimation, and then transmit the local state estimation value to the remote state estimator. Therefore, we use ${\tilde{x}}_{i}\left(k\right)$ and ${\tilde{F}}_{i}\left(k\right)$ to represent the *i*th sensor’s local minimum mean-squared error (MMSE) estimate of the state and the corresponding error covariance [32]:

(3) $$\begin{array}{ccc} {\tilde{x}}_{i}\left(k\right) & = & E[x_{i}\left(k\right)|y_{i}\left(1\right),y_{i}\left(2\right),...,y_{i}\left(k\right)],\\ {\tilde{F}}_{i}\left(k\right) & = & E[\left(x_{i}\left(k\right)-{\tilde{x}}_{i}\left(k\right)\right){\left(x_{i}\left(k\right)-{\tilde{x}}_{i}\left(k\right)\right)}^{T} \end{array}$$

(4) $$\begin{array}{ccc} & & |y_{i}\left(1\right),y_{i}\left(2\right),...,y_{i}\left(k\right)], \end{array}$$

which can be calculated based on a standard Kalman filter:

$$\begin{array}{ccc} {\tilde{x}}_{i}\left(k|k-1\right) & = & A_{i}{\tilde{x}}_{i}\left(k-1\right),\\ {\tilde{F}}_{i}\left(k|k-1\right) & = & A_{i}{\tilde{F}}_{i}\left(k-1\right)A_{i}^{T}+Q_{i},\\ K_{i}\left(k\right) & = & {\tilde{F}}_{i}\left(k|k-1\right)C_{i}^{T}{\left[C_{i}{\tilde{F}}_{i}\left(k|k-1\right)C_{i}^{T}+R_{i}\right]}^{-1},\\ {\tilde{x}}_{i}\left(k\right) & = & A_{i}{\tilde{x}}_{i}\left(k-1\right)+K_{i}\left(k\right)\left(y_{i}\left(k\right)-C_{i}A_{i}{\tilde{x}}_{i}\left(k-1\right)\right),\\ {\tilde{F}}_{i}\left(k\right) & = & \left(I-K_{i}\left(k\right)C_{i}\right){\tilde{F}}_{i}\left(k\right), \end{array}$$

where

$$\begin{array}{ccc} {\tilde{x}}_{i}\left(k|k-1\right) & = & E[x_{i}\left(k\right)|y_{i}\left(1\right),y_{i}\left(2\right),...,y_{i}\left(k-1\right)],\\ {\tilde{F}}_{i}\left(k|k-1\right) & = & E[\left(x_{i}\left(k\right)-{\tilde{x}}_{i}\left(k\right)\right){\left(x_{i}\left(k\right)-{\tilde{x}}_{i}\left(k\right)\right)}^{T}\\ & & |y_{i}\left(1\right),y_{i}\left(2\right),...,y_{i}\left(k-1\right)], \end{array}$$

$K_{i}\left(k\right)$ is the gain of the Kalman filter and the initial condition is ${\tilde{x}}_{i}\left(0\right)=0$.

For ease of representation, we can also define the Lyapunov and Riccati operators *h*, $\tilde{g}$: $S_{+}^{n}$→$S_{+}^{n}$ as

(5) $$\begin{array}{ccc} h_{i}\left(X\right) & ≜ & A_{i}XA_{i}^{T}+Q_{i}, \end{array}$$

(6) $$\begin{array}{ccc} {\tilde{g}}_{i}\left(X\right) & ≜ & X-XC_{i}^{T}{\left[C_{i}XC_{i}^{T}\right]}^{-1}C_{i}X, \end{array}$$

(7) $$\begin{array}{ccc} h_{i}^{k}\left(X\right) & ≜ & \underset{k\;\mathrm{times}}{\underset{︸}{h_{i}\circ h_{i}\circ \cdot \cdot \cdot \circ h_{i}\circ h_{i}}}\left(X\right). \end{array}$$

Under the assumptions of detectability and stability, it has been shown that the posterior estimation error covariance matrix of the Kalman filter converges exponentially from any initial condition to a unique value $\tilde{F}$, [33], i.e., ${\tilde{F}}_{i}\left(k\right)={\tilde{F}}_{i}$, k≥1, which ${\tilde{F}}_{i}$ is the steady-state error covariance, which is determined by the unique positive semi-definite solution of ${\tilde{g}}_{i}\circ h_{i}\left(X\right)$=*X* [34].

 **Lemma 1** ([35]). *For $0\leq \xi_{1}\leq \xi_{2}$, it has*

(8) $$Tr\left\{\tilde{F}\right\}\leq Tr\left\{h_{i}^{\xi_{1}}\left(\tilde{F}\right)\right\}\leq Tr\left\{h_{i}^{\xi_{2}}\left(\tilde{F}\right)\right\}.$$

### 2.2. Attack Model Based on SINR

To simulate random data loss due to fading and interference, we assume that the communication between the sensor and the remote estimator or the eavesdropper is via an Additive White Gaussian Noise (AWGN) channel using quadrature amplitude modulation (QAM). Data packets sent by the sensor are quantized and mapped to QAM symbols. Then, digital communication theory reveals the relationship between symbol error rate (SER) and SINR as follows [5,36]:

(9) $$SER_{i}^{℘}=2Q\left(\sqrt{\alpha SINR_{i}}\right)$$

where $Q\left(x\right)=\frac{1}{\sqrt{2\pi }}\int_{x}^{\infty }e^{-t^{2}/2}dt$, ℘∈{e,a} and α>0 is a parameter. ℘=e represents the remote estimator side, ℘=a indicates the eavesdropper side.

Considering the remote estimator side first, the channel SINR for the remote estimator at time *k* is [24],

(10) $$SINR_{i}^{e}=\frac{\Phi_{i}p_{i}\left(k\right)}{\sigma_{i,e}^{2}}$$

where $\Phi_{i}$ is the channel gain of the *i*th communication channel between the sensor and the remote estimator. $p_{i}\left(k\right)\geq 0$ is the transmission power for the QAM symbol used by sensor *i* at time *k* and $\sigma_{i,e}^{2}$ is the AWGN power of the *i*th channel between the sensor and the remote estimator. Define a random variable $\zeta_{i}\left(k\right)\in \left\{0,1\right\}$ as to whether the remote estimator successfully receives the information at time *k*, i.e.,

$$\zeta_{i}^{e}\left(k\right)=\left\{\begin{array}{cc} 0, & \mathrm{otherwise}\;(\mathrm{regarded}\;\mathrm{as}\;\mathrm{dropout})\\ 1, & \mathrm{if}\;{\tilde{x}}_{i}\left(k\right)\;\mathrm{is}\;\mathrm{successfully}\;\mathrm{received}\;\mathrm{by}\;\mathrm{the}\;\mathrm{estimator}. \end{array}\right.$$

Then, the packet arrival rate of the remote estimator is

(11) $$\begin{array}{cc} \lambda_{i}^{e}\left(k\right) & =P\left\{\zeta_{i}^{e}\left(k\right)=1\right\}=1-SER_{i}^{e}\\ & =1-2Q(\sqrt{\alpha \frac{\Phi_{i}p_{i}\left(k\right)}{\sigma_{i,e}^{2}}}). \end{array}$$

Secondly, considering the eavesdropper side, we can know that the SINR of the channel at the eavesdropper side at time *k* can be expressed as

(12) $$SINR_{i}^{a}=\frac{\Psi_{i}p_{i}\left(k\right)}{a_{i}\left(k\right)+\sigma_{i,a}^{2}}$$

where $\Psi_{i}$ is the channel gain of the *i*th communication channel between the sensor and the eavesdropper. $a_{i}\left(k\right)$ indicates the attack power to the *i*th channel launched by the eavesdropper. $\sigma_{i,a}^{2}$ is the AWGN power of the channel between the sensor and the eavesdropper. Similarly, we use a binary random variable $\zeta_{i}^{a}\left(k\right)$ to indicate whether the eavesdropper is successful in eavesdropping, i.e.,

$$\zeta_{i}^{a}\left(k\right)=\left\{\begin{array}{cc} 0, & \mathrm{otherwise}\\ 1, & \mathrm{if}\;{\tilde{x}}_{i}\left(k\right)\;\mathrm{is}\;\mathrm{successfully}\;\mathrm{eavesdropped}\;\mathrm{by}\;\mathrm{the}\;\mathrm{eavesdropper} \end{array}\right.$$

Then, the probability that an eavesdropper can successfully eavesdrop is:

(13) $$\begin{array}{cc} \lambda_{i}^{a}\left(k\right) & =P\left\{\zeta_{i}^{a}\left(k\right)=1\right\}=1-SER_{i}^{a}\\ & =1-2Q(\sqrt{\alpha \frac{\Psi_{i}p_{i}\left(k\right)}{a_{i}\left(k\right)+\sigma_{i,a}^{2}}}). \end{array}$$

Hypothetical processes $\zeta_{i}^{e}\left(k\right)$ and $\zeta_{i}^{a}\left(k\right)$ are independent of each other.

### 2.3. Remote State Estimation

With the local estimate received by the remote estimator, we can determine the MMSE state estimate ${\hat{x}}_{i}^{e}\left(k\right)$ and the corresponding estimation error covariance $F_{i}^{e}\left(k\right)$ of the remote estimator at time k, where ${\hat{x}}_{i}^{e}\left(k\right)$ and $F_{i}^{e}\left(k\right)$ are obtained by the following iterative process:

(14) $${\hat{x}}_{i}^{e}\left(k\right)=\left\{\begin{array}{cccc} & A_{i}{\hat{x}}_{i}^{e}\left(k-1\right), & & \mathrm{otherwise},\\ & {\tilde{x}}_{i}\left(k\right), & & \mathrm{if}\zeta_{i}^{e}\left(k\right)=1, \end{array}\right.$$

(15) $$F_{i}^{e}\left(k\right)=\left\{\begin{array}{cccc} & h_{i}\left(F_{i}^{e}\left(k-1\right)\right), & & \mathrm{otherwise},\\ & \tilde{F_{i}}, & & \mathrm{if}\zeta_{i}^{e}\left(k\right)=1. \end{array}\right.$$

Similarly, denote ${\hat{x}}_{i}^{a}\left(k\right)$ and $F_{i}^{e}\left(k\right)$ as the MMSE state estimation and corresponding error covariance of the eavesdropper at time k, then ${\hat{x}}_{i}^{a}\left(k\right)$ and $F_{i}^{a}\left(k\right)$ can be expressed as

(16) $${\hat{x}}_{i}^{a}\left(k\right)=\left\{\begin{array}{cccc} & A_{i}{\hat{x}}_{i}^{a}\left(k-1\right), & & \mathrm{otherwise},\\ & {\tilde{x}}_{i}\left(k\right), & & \mathrm{if}\zeta_{i}^{a}\left(k\right)=1, \end{array}\right.$$

(17) $$F_{i}^{a}\left(k\right)=\left\{\begin{array}{cccc} & h_{i}\left(F_{i}^{a}\left(k-1\right)\right), & & \mathrm{otherwise},\\ & \tilde{F_{i}}, & & \mathrm{if}\zeta_{i}^{a}\left(k\right)=1. \end{array}\right.$$

Therefore,

(18) $$\begin{array}{ccc} E[F_{i}^{e}\left(k\right)] & = & \lambda_{i}^{e}\left(k\right){\tilde{F}}_{i}+\left(1-\lambda_{i}^{e}\left(k\right)\right)h_{i}\left(F_{i}^{e}\left(k-1\right)\right), \end{array}$$

(19) $$\begin{array}{ccc} E[F_{i}^{a}\left(k\right)] & = & \lambda_{i}^{a}\left(k\right){\tilde{F}}_{i}+\left(1-\lambda_{i}^{a}\left(k\right)\right)h_{i}\left(F_{i}^{a}\left(k-1\right)\right). \end{array}$$

Define **S** ≜$\left\{{\tilde{F}}_{i},h_{i}\left({\tilde{F}}_{i}\right),h_{i}^{2}\left({\tilde{F}}_{i}\right)...\right\}$, it is composed of all possible values of $F_{i}^{e}\left(k\right)$ and $F_{i}^{a}\left(k\right)$.

### 2.4. Problem Formulation

Specifically, we consider the following problem: from the perspective of the eavesdropper, in an infinite time horizon, the eavesdropper finds the optimal attack allocation under the condition of limited energy to maximize the state estimation error of the eavesdropper, i.e.,

 **Problem 1.**
(20) $$\begin{array}{ccc} & & \max_{\theta }J\left(\pi \right), \end{array}$$

(21) $$\begin{array}{ccc} s.t. & & \sum_{i=1}^{N}a_{i}\left(k\right)\leq \bar{a} \end{array}$$

* where $\pi =\{\left(a_{1}\left(1\right),a_{2}\left(1\right),\ldots ,a_{N}\left(1\right)\right),\left(a_{1}\left(2\right),a_{2}\left(2\right),\ldots ,a_{N}\left(2\right)\right),\ldots \}$ is an admissible attack policy with the attack power using a time-step 1,2,..... $\bar{a}$ is an energy constraint for the attacker at each time. The infinite horizon average expectation of the remote estimation error covariance can be expressed by the following formula:*

(22) $$\begin{array}{c} J\left(\pi \right)=\underset{T\to \infty }{\lim\inf}\frac{1}{T+1}\left[E\sum_{k=0}^{T}\sum_{i=1}^{N}tr\left(F_{i}^{a}\left(k\right)\right)\right] \end{array}$$

## 3. Optimal Attack Schedule

In this section, we formulate Problem 1 as a discrete time MDP to solve. In addition, we also give an algorithm for searching the optimal eavesdropping attack strategy.

### 3.1. MDP Formulation

For the convenience of notation, denote $\tau_{i}^{e}\left(k\right)$ (or $\tau_{i}^{a}\left(k\right)$) as the holding time from the estimator (or eavesdropper) to the continuous successful acquisition of data at time *k*, that is, the duration from the last successful transmission time to time *k*, which can be expressed by the following formula:

(23) $$\begin{array}{ccc} \tau_{i}^{e}\left(k\right) & = & k-\max\{k^{*}:\zeta_{i}^{e}\left(k^{*}\right)=1,0\leq k^{*}\leq k\}, \end{array}$$

(24) $$\begin{array}{ccc} \tau_{i}^{a}\left(k\right) & = & k-\max\{k^{*}:\zeta_{i}^{a}\left(k^{*}\right)=1,0\leq k^{*}\leq k\}. \end{array}$$

Obviously, $\tau_{i}^{e}\left(k\right)\in S_{i}^{e}=N$ (or $\tau_{i}^{a}\left(k\right)\in S_{i}^{a}=N$), then we can get:

(25) $$\begin{array}{ccc} F_{i}^{e}\left(k\right) & = & h^{\tau_{i}^{e}\left(k\right)}\left(\tilde{F}\right), \end{array}$$

(26) $$\begin{array}{ccc} F_{i}^{a}\left(k\right) & = & h^{\tau_{i}^{a}\left(k\right)}\left(\tilde{F}\right). \end{array}$$

Using MDP to describe the dynamic process of CPS under eavesdropping attacks, MDP is expressed mathematically as 〈S,A,P,r(·)〉, and the specific elements are as follows.

**State space**: let $s_{i}\left(k\right)=\left(\tau_{i}^{e}\left(k-1\right),\tau_{i}^{a}\left(k-1\right)\right)\in S_{i}^{e}\times S_{i}^{a}=N^{2}$, where $\tau_{i}^{e}\left(k-1\right)$ and $\tau_{i}^{a}\left(k-1\right)$ can be considered as the state of process *i* at time k−1 at the remote estimator side and eavesdropper side, respectively. The state at time *k* is defined as $s\left(k\right)=(s_{1}\left(k\right),s_{2}\left(k\right),\ldots ,s_{N}\left(k\right))$, and its value range is a countable state space $S_{i}≜S_{i}^{e}\times S_{i}^{a}$. Let $S=\{S_{1},\ldots ,S_{N}\}$.

**Action space**: we can know the action space is defined as $A≜\{a_{1}\left(k\right),a_{2}\left(k\right),\ldots ,a_{N}\left(k\right)\}$, where $a_{i}\left(k\right)\in \left(0,a_{i}^{\left(1\right)},a_{i}^{\left(2\right)},\ldots ,a_{i}^{\left(l_{i}\right)}\right)$, i∈{1,2,…,N}, $a_{i}^{\left(l_{i}\right)}$ is the maximum attack power to channel *i*. Thus, the action is $a\left(k\right)≜\{a_{1}\left(k\right),a_{2}\left(k\right),\ldots ,a_{N}\left(k\right)\}\in A$.

**Transition probability**: let the state transition introduction matrix at time *k* be $P_{i}\left(s_{i}\left(k+1\right)|s_{i}\left(k\right),a_{i}\left(k\right)\right)$, which represents the probability of the state changing from $s_{i}\left(k\right)$ to $s_{i}\left(k+1\right)$ under action $a_{i}\left(k\right)$, where $s_{i}\left(k\right),s_{i}\left(k+1\right)\in S$, $a_{i}\left(k\right)\in A$. For simplicity, let the state at time *k* be $s_{i}\left(k\right)=\left(j_{1},j_{2}\right)$. Then, the state transition probability matrix is as follows:

(27) $$\begin{array}{c} P_{i}\left(s_{i}\left(k+1\right)|s_{i}\left(k\right),a_{i}\left(k\right)\right)= \end{array}\;\left\{\begin{array}{cccc} & \lambda_{i}^{e}\left(k\right)\lambda_{i}^{a}\left(k\right), & & \mathrm{if}s_{i}\left(k+1\right)=\left(0,0\right),\\ & \lambda_{i}^{e}\left(k\right)\left(1-\lambda_{i}^{a}\left(k\right)\right), & & \mathrm{if}s_{i}\left(k+1\right)=\left(0,j_{2}+1\right),\\ & \left(1-\lambda_{i}^{e}\left(k\right)\right)\lambda_{i}^{a}\left(k\right), & & \mathrm{if}s_{i}\left(k+1\right)=\left(j_{1}+1,0\right),\\ & \left(1-\lambda_{i}^{e}\left(k\right)\right)\left(1-\lambda_{i}^{a}\left(k\right)\right), & & \mathrm{if}s_{i}\left(k+1\right)=\left(j_{1}+1,j_{2}+1\right). \end{array}\right.$$

**Payoff functions**: let r(·) be the immediate cost function and define it as:

(28) $$\begin{array}{ccc} r(s(k),a(k)) & ≜ & \sum_{i=1}^{N}Tr\left(F_{i}^{a}\left(k\right)\right) \end{array}$$

Obviously, the single-stage reward at time *k* is independent of the action behavior and only depends on the current state.

Note that the random decision rule of the eavesdropper is a mixed strategy sequence $\pi =\{\left(a_{1}\left(1\right),a_{2}\left(1\right),\ldots ,a_{N}\left(1\right)\right),\left(a_{1}\left(2\right),a_{2}\left(2\right),\ldots ,a_{N}\left(2\right)\right),\ldots \}$, where π is the random kernel from H to A and definition Π is the set of all these feasible strategies. Based on the process state s(k), the attacker chooses action a(k)=a(s(k)), π={(a(s(1)),…,a(s(k)),…}. Then, for the initial state s(0)=s∈S, we can get the sum of expected reward r(k) following the action strategy π∈Π:

(29) $$J\left(s,\pi \right)=\underset{T\to \infty }{\lim\inf}\frac{1}{T+1}E\left[\sum_{k=0}^{T}r\left(s\left(k\right),a\left(k\right)\right)\right]$$

and its optimal value $J^{*}\left(s\right)$ is

(30) $$J^{*}\left(s\right)=\arg\max_{\pi \in \Pi }J\left(s,\pi \right).$$

Define the average value function under policy π∈Π as the function *V*: S→R. Therefore, we can get the following theorem.

 **Theorem 1.** According to the MDP theory, we can obtain the optimal value $J^{*}\left(s\right)$ by solving the following optimality (Bellman) equation:

(31) $$\begin{array}{cc} J^{*}\left(s\right)+V\left(s\right) & =\max_{a(k)\in A}\{r\left(s,a\right)\\ & +\sum_{s(k+1)\in S}\prod_{i=1}^{N}P_{i}\left(s_{i}\left(k+1\right)|s_{i}\left(k\right),a_{i}\left(k\right)\right)V\left(s\left(k+1\right)\right)\} \end{array}$$

*where s = $(s_{1},\ldots ,s_{N})\in S$ is the initial state.*

*The optimal attack strategies of the eavesdropper is:*

(32) $$\begin{array}{cc} a^{*}\left(s\left(k\right)\right) & =\arg\max_{a(k)\in A}\{r\left(s\left(k\right),a\left(k\right)\right)\\ & +\sum_{s(k+1)\in S}\prod_{i=1}^{N}P_{i}\left(s_{i}\left(k+1\right)|s_{i}\left(k\right),a_{i}\left(k\right)\right)J^{*}\left(s\left(k+1\right)\right)\} \end{array}$$

**Proof (Proof of Theorem 1).**  According to the eighth chapter in reference [37], Theorem 1 can be obtained by introducing our state transition probability matrix (27) and immediate cost function (28).

From Equation (8.4.2) in [37], we can get the following equation:

$$\begin{array}{cc} J^{*}\left(s\right)+V\left(s\right) & =\max_{a(k)\in A}\left\{r_{\pi_{1}}+\sum_{k=1}^{\infty }\prod_{i=1}^{k-1}P_{\pi_{i}}r_{\pi_{k}}\right\}\\ & =\max_{a(k)\in A}\left\{r_{\pi_{1}}+P_{\pi_{1}}r_{\pi_{2}}+P_{\pi_{1}}P_{\pi_{2}}r_{\pi_{3}}+\cdot \cdot \cdot \right\}\\ & =\max_{a(k)\in A}\left\{r_{\pi_{1}}+P_{\pi_{1}}\left[r_{\pi_{2}}+P_{\pi_{2}}r_{\pi_{3}}+\cdot \cdot \cdot \right]\right\}\\ & =\max_{a(k)\in A}\left\{r_{\pi_{1}}+P_{\pi_{1}}V\left(s\left(k+1\right)\right)\right\} \end{array}$$

where $\pi_{k}$ is the strategy of the time *k*. *r* and P are abbreviations. Many decision rules are contained in historical strategies. So, $r_{\pi_{1}}$ and $P_{\pi_{1}}$ can be decomposed into the following formula:

(33) $$\begin{array}{ccc} r_{\pi_{1}} & = & r(s,a),\\ P_{\pi_{1}} & = & \sum_{s(k+1)\in S}\prod_{i=1}^{N}P_{i}\left(s_{i}\left(k+1\right)|s_{i}\left(k\right),a_{i}\left(k\right)\right). \end{array}$$

Therefore, we can get the following:

(34) $$\begin{array}{cc} J^{*}\left(s\right)+V\left(s\right) & =\max_{a(k)\in A}\left\{r_{\pi_{1}}+P_{\pi_{1}}V\left(s\left(k+1\right)\right)\right\}\\ & =\max_{a(k)\in A}\{r\left(s,a\right)\\ & +\sum_{s(k+1)\in S}\prod_{i=1}^{N}P_{i}\left(s_{i}\left(k+1\right)|s_{i}\left(k\right),a_{i}\left(k\right)\right)V\left(s\left(k+1\right)\right)\} \end{array}$$

Then, we rewrite the finite-horizon optimality Equation (4.5.1) in [37] as

(35) $$\begin{array}{cc} a^{*}\left(s\left(k\right)\right) & =\arg\max_{a(k)\in A}\{r\left(s\left(k\right),a\left(k\right)\right)\\ & +\sum_{s(k+1)\in S}\prod_{i=1}^{N}P_{i}\left(s_{i}\left(k+1\right)|s_{i}\left(k\right),a_{i}\left(k\right)\right)J^{*}\left(s\left(k+1\right)\right)\} \end{array}$$

Thus, we can get the optimality (Bellman) Equation (31) and the optimal attack strategies of the eavesdropper (32). So, Theorem 1 is proved.    □

 **Remark 1.** *It should be noted that for finite MDP, the action a(k) taken at time k is non-stationary and depends on the current state at time k.*

 **Remark 2.** *We can get the optimal attack energy allocation strategy of (29) by using the optimality (Bellman) Equation (31); in addition, the optimal strategy is statically deterministic, which helps us to find out the structural characteristics of the optimal allocation strategy.*

### 3.2. Policy Iteration Algorithm

MDP proposed in this paper has infinite state space. However, according to the characteristics of state transition in the system model, we can find that when the eavesdropper’s attack energy is limited, the transition rule can effectively limit the system state in a limited time range. Therefore, in the MDP proposed in this paper, although it has infinite state space, we can treat it as an MDP with a finite time domain. This is convenient for us to design the algorithm of the optimal attack strategy.

In a finite time domain, the solution of the optimal equation is the optimal quality function from the decision time *k* to the decision time *T* at the end of the process. Based on the MDP problem constructed above, we provide a specific backward induction algorithm to solve it and provide the optimal attack strategy, i.e., Algorithm 1.

In Algorithm 1, we first calculate ${\tilde{F}}_{i}$, the packet rate $\lambda_{i}^{e}\left(k\right),\lambda_{i}^{a}\left(k\right)$ and the hold time $\tau_{i}^{e}\left(k\right)$, $\tau_{i}^{a}\left(k\right)$ in step 1 and calculate the state transition matrix $P_{i}$ in step 2. Then, in step 3, set all k=0 and for all s(k)∈S, compute $J^{*}\left(s\right)+V\left(s\right)$ by (31). Next, in step 4, we set k←k−1, initialize s(k)=s(k+T). In step 5, let s(k)∈S, compute $J^{*}\left(s\right)$ by (31) and $a^{*}\left(s\left(k\right)\right)$ by (32). We assess that the best action 1 is found for state s(k), so in step 6, if $a^{*}\left(s\left(k\right)\right)=1$, then for all s(k+1)∈S, let (34). After this, let s(k)←s(k+T), and go to Step 5. Otherwise, let s(k)←s(k+1), go to Step 5. Finally, in step 7, if k=0, output $J_{T}^{*}\left(s\right)$ and $\pi^{*}=\left(a^{*}\left(0\right),a^{*}\left(1\right),...,a^{*}\left(T-1\right)\right)$. Otherwise, go to Step 4.
**Algorithm 1** Backward induction algorithm for optimal allocation strategy**Require: **$A_{i},C_{i},Q_{i},R_{i},p_{i}\left(k\right),\sigma_{e},\sigma_{a},\bar{a},T,S,A,s$.
**Ensure:** The optimal value $J_{T}^{*}\left(s\right)$; optimal deterministic Markov policy $\pi^{*}$
 **Step 1:** Calculate ${\tilde{F}}_{i}$, the packet rate $\lambda_{i}^{e}\left(k\right),\lambda_{i}^{a}\left(k\right)$ and the holding time $\tau_{i}^{e}\left(k\right)$, $\tau_{i}^{a}\left(k\right)$
 **Step 2:** Calculate state transition matrix $P_{i}$
 **Step 3:** Set all k=0 and for all s(k)∈S, compute $J^{*}\left(s\right)+V\left(s\right)$ by (31). **Step 4:** Set k←k−1, initialize s(k)=s(k+T). **Step 5:** Let s(k)∈S, compute
$$\begin{array}{cc} J^{*}\left(s\right)+V\left(s\right) & =\max_{a(k)\in A}\{r\left(s,a\right)\\ & +\sum_{s(k+1)\in S}\prod_{i=1}^{N}P_{i}(s_{i}\left(k+1\right)\\ & |s_{i}\left(k\right),a_{i}\left(k\right))V\left(s\left(k+1\right)\right)\} \end{array}$$
$$\begin{array}{cc} a^{*}\left(s\left(k\right)\right) & =\arg\max_{a(k)\in A}\{r\left(s\left(k\right),a\left(k\right)\right)\\ & +\sum_{s(k+1)\in S}\prod_{i=1}^{N}P_{i}\left(s_{i}(k+1)|s_{i}(k),a_{i}(k)\right)J^{*}(s(k+1))\}. \end{array}$$ **Step 6:** If $a^{*}\left(s\left(k\right)\right)=1$, then, for all s(k+1)∈S, let
(36)$$\begin{array}{c} \begin{array}{cc} a^{*}\left(s\left(k+1\right)\right)\\ =1,J^{*}\left(s\left(k+1\right)\right)+V(s\left(k+1\right))=\\ r(s(k+1),1) & +\sum_{s(k+2)\in S}\prod_{i=1}^{N}P_{i}\left(s_{i}\left(k+2\right)|s_{i}\left(k+1\right),1\right)V\left(s\left(k+2\right)\right) \end{array} \end{array}$$ let s(k)←s(k+T), and go to **Step 5**. Otherwise, let s(k)←s(k+1), go to **Step 5**. **Step 7:** If k=0, then output $J_{T}^{*}\left(s\right)$ and $\pi^{*}=\left(a^{*}\left(0\right),a^{*}\left(1\right),...,a^{*}\left(T-1\right)\right)$. Otherwise, go to **Step 4**.

 **Remark 3.** In the above Algorithm 1, it is assumed that $a^{*}\left(s\left(k\right)\right)=1$ in order to reduce the calculation cost and complexity compared with the traditional algorithm.

 **Remark 4.** *We can derive the state estimation of the eavesdropper each time to ensure the feasibility of Algorithm 1.*

## 4. Discussion and Illustrative Example

In this section, according to the above MDP model, we can provide some numerical simulations to show the optimal attack energy allocation strategy of problem 1. Consider systems (1) and (2) with β=0.5. The parameters of the systems and channels are shown in Table 1.

Suppose the energy constraint is $\bar{a}=10$. In Table 1, $a_{1}^{\left(1\right)}=0,\;a_{1}^{\left(2\right)}=5,$$a_{1}^{\left(3\right)}=7,\;a_{1}^{\left(4\right)}=10$ indicates that the possible maximum attack power of channel 1 is 0, 5, 7 and 10, respectively, and another 0 means that there is no attack. $a_{2}^{\left(1\right)}=0,$$a_{2}^{\left(2\right)}=3,\;a_{2}^{\left(3\right)}=5,\;a_{2}^{\left(4\right)}=10$ in the same way. According to the number of existing systems and the number of channels that eavesdrop at the same time, we can divide them into the following three methods, such as numerical simulation. We use the Algorithm 1 to calculate the optimal attack energy allocation strategy and the optimal average return.

### 4.1. Single System

Let us first consider the case where there is only one system. When there is only one system, we consider the optimal attack energy allocation strategy when attacking this channel at different times. We can use the data in Table 1 about Sensor 1. The strategy set for the eavesdropper is {(0,0),(10,0),(0,10),(5,5)}, where (0,0) means no attack, (10,0) means use energy 10 to attack at the first moment and not to attack at the second moment and (0,10) and (5,5) have a similar meaning. Assume that the transmission power of the sensor is p(k)=0.6. Through the calculation, the error covariance of the steady state estimation is obtained, i.e., $\tilde{F}=1.9755$. Assume that the AWGN power of the channel between the sensor and the estimator is $\sigma_{e}^{2}=0.3$, and the AWGN power of the channel between the sensor and the eavesdropper is $\sigma_{a}^{2}=0.5$. This paper studies the infinite time domain, but in order to simplify the calculation, we use the truncated set $N_{0}=\left\{0,1,...,16\right\}$. The optimal strategy is calculated through Algorithm 1.

**Figure 2:**
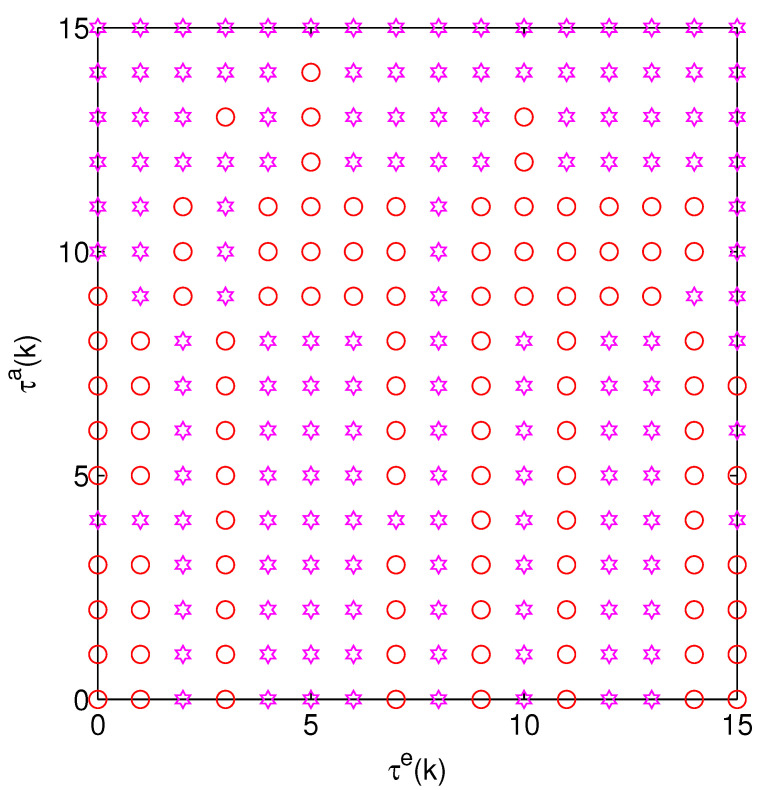
A single system’s optimal energy allocation.

In Figure 2, we use $\tau^{e}\left(k\right)$ and $\tau^{a}\left(k\right)$ to represent the state of the optimal strategy and the optimal strategy is shown, where the purple and red symbols represent policy 1 (policy 1 = (0, 0)) and policy 2 (policy 2 = (0, 10)).

### 4.2. Dual System (Not Attacking or Attacking One Channel)

Consider that when there are two systems, neither of the two channels can attack or can only attack one channel.In the numerical solution, we consider the data in Table 1 and Table 2 and use a truncated set $h_{\left(i\right)}^{35}\left({\tilde{F}}_{\left(i\right)}\right)$. We can assume that the eavesdropper’s strategy set is {(0,0),(5,0),(7,0),(10,0),(0,3),(0,5),(0,10)}, i.e., Table 3. Where (0,0) indicates that there is no attack on both channels. (5,0),(7,0),(10,0) means that channel 1 is attacked with energy 5, 7 and 10, respectively, and channel 2 is not attacked; (0,3),(0,5),(0,10) has the same meaning. Through our calculations, we can get ${\tilde{F}}_{1}=1.9755$, ${\tilde{F}}_{2}=1.6805$. $\Phi_{i}$ and $\Psi_{i}$ we take as random numbers.

**Figure 3:**
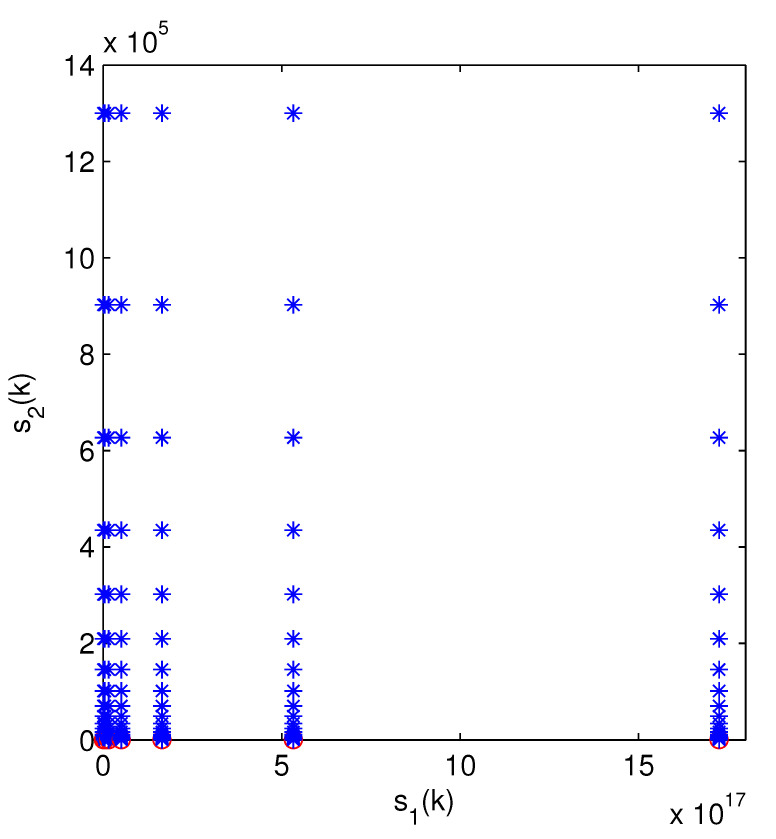
Optimal action of state $s=(s_{1},s_{2})$ (not attacking or attacking one channel), where the blue stars and red circles represent the actions (0,10) and (10,0), respectively.

**Figure 4:**
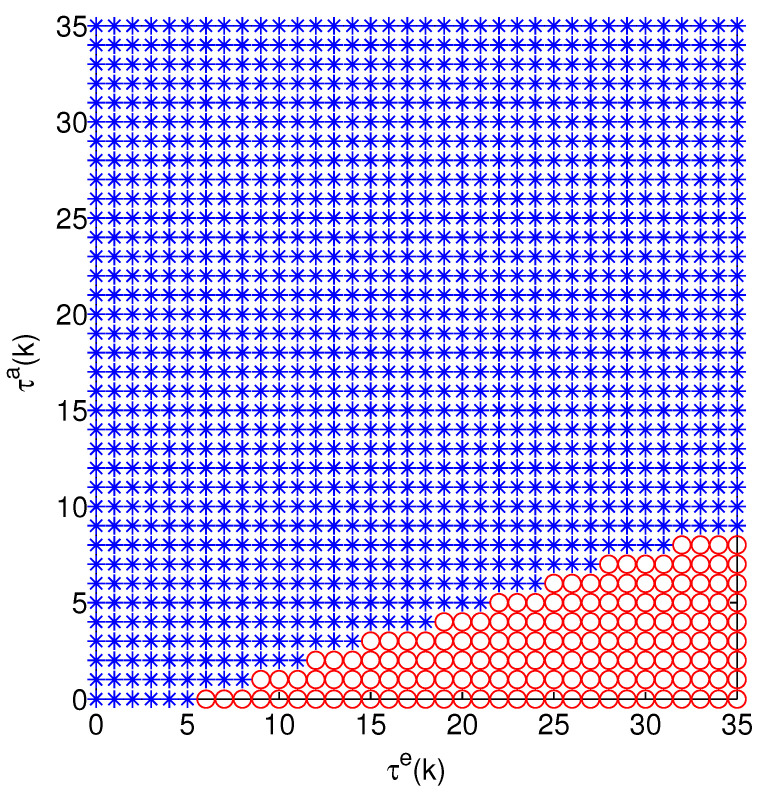
Optimal action of ($\tau^{e}\left(k\right),\tau^{a}\left(k\right)$). The meaning of circles and stars is the same as in Figure 3.

The optimal attack energy allocation strategy when there are two systems, neither of which attack or only one attacks, is shown in Figure 3 and Figure 4. In Figure 3, with an increase of $s_{1}\left(k\right)$, the optimal strategies cannot be clearly identified. Therefore, we use $\tau^{e}\left(k\right)$ and $\tau^{a}\left(k\right)$ in Figure 4 to represent the state of the optimal strategies. From Figure 4, we can see that the optimal action includes (0,10) and (10,0).

### 4.3. Dual System (Not Attacking, Attacking One Channel or Attacking Two Channels)

Similarly, in this section, two systems are also considered, and we can consider three situations: no attack, only one channel attack and two channels attack at the same time. We can assume that the eavesdropper’s strategy set is {(0,0),(5,0),(10,0),(0,5),(0,10),(5,5),(7,3)}, i.e., Table 4. Where (0,0) indicates that there is no attack on both channels. (5,0),(10,0) means that channel 1 is attacked with energy 5 and 10, respectively, and channel 2 is not attacked; (0,5),(0,10) has the same meaning. (5,5) represents an attack on channel 1 and channel 2 using energy 5 and energy 5, respectively; (7,3) has the same meaning. In the numerical solution, we consider the data in Table 1 and Table 2 and use truncated set $h_{\left(i\right)}^{35}\left({\tilde{F}}_{\left(i\right)}\right)$. $\Phi_{i}$ and $\Psi_{i}$ we take as random numbers. Through our calculations, we can get ${\tilde{F}}_{1}=1.9755$, ${\tilde{F}}_{2}=1.6805$.

**Figure 5:**
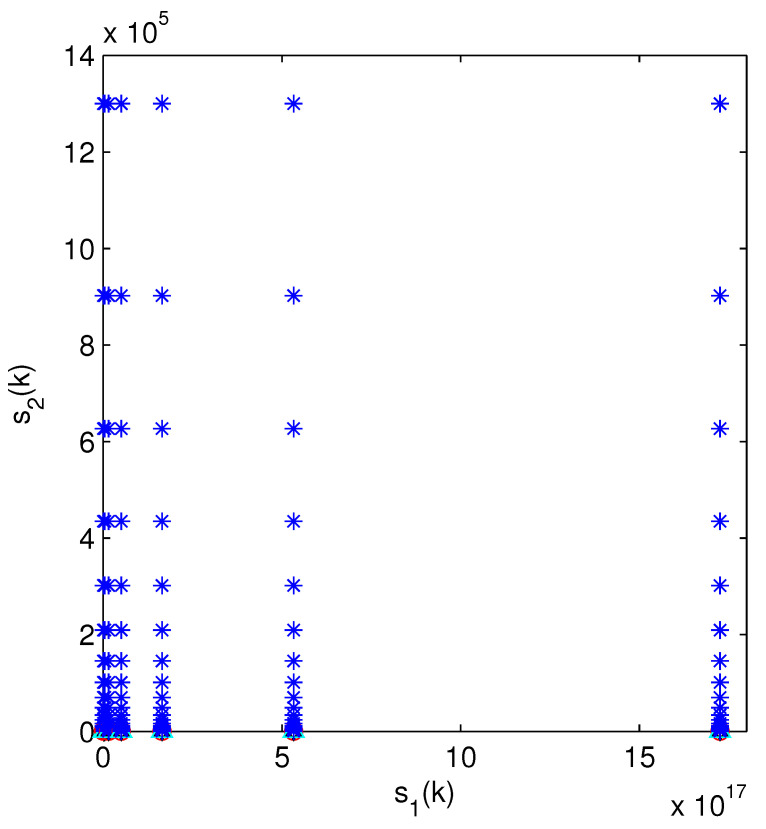
Optimal action of state $s=(s_{1},s_{2})$ (not attacking, attacking one channel or attacking two channels), where the blue stars, green triangles, black pentagrams and red circles represent the actions (0,10), (5,5), (7,3) and (10,0), respectively.

**Figure 6:**
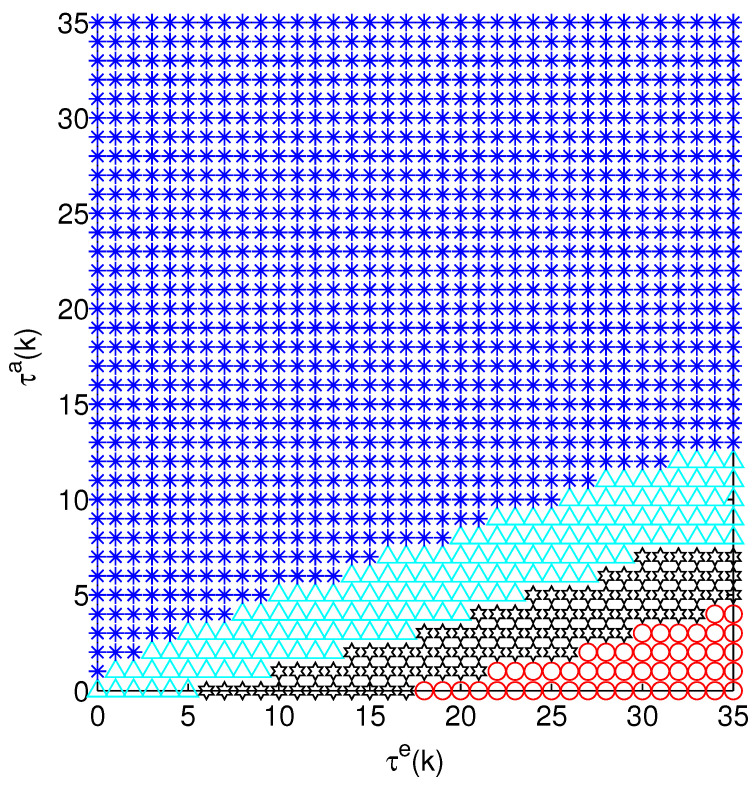
Optimal action of ($\tau^{e}\left(k\right),\tau^{a}\left(k\right)$). The meaning of circles, triangles, pentagrams and stars is the same as in Figure 5.

Figure 5 and Figure 6 show the optimal attack energy allocation strategy when there are two systems, in which two channels are not attacked, only one channel is attacked and both channels are attacked. In Figure 5, with an increase of $s_{1}\left(k\right)$, the optimal strategies cannot be clearly identified. Therefore, we use $\tau^{e}\left(k\right)$ and $\tau^{a}\left(k\right)$ in Figure 6 to represent the state of the optimal strategies. From Figure 6, we can see that the optimal action includes (0,10), (5,5), (7,3) and (10,0).

## 5. Conclusions

The optimal attack energy allocation of multi-system remote state estimation in CPS is studied when eavesdroppers exist in an infinite time domain. Based on the channel SINR, a wireless communication model is constructed. When the eavesdropper’s energy is limited, the optimal value of the eavesdropper and the optimal attack energy allocation strategy are found by using MDP theory. Finally, the research results are numerically simulated. According to the theoretical analysis and the numerical analysis, we can draw the following conclusions: in a multi-system, the optimal energy allocation strategy is to choose a higher attack energy to attack the channel when the estimation error covariance at the eavesdropper is large, and choose a lower attack energy to attack the channel when the estimation error covariance is small. And, we can see that the optimal attack strategy has an obvious threshold structure. In the future, we will prove the threshold structure of the optimal allocation strategy and study the situation when the detector exists.

## Figures and Tables

**Table 1:** Parameters for sensors and attack power.

Sensor 1								Sensor 2							
**Process 1**				**Attack Power**				**Process 2**				**Attack Power**			
$A_{1}$	$C_{1}$	$Q_{1}$	$R_{1}$	$a_{1}^{\left(1\right)}$	$a_{1}^{\left(2\right)}$	$a_{1}^{\left(3\right)}$	$a_{1}^{\left(4\right)}$	$A_{2}$	$C_{2}$	$Q_{2}$	$R_{2}$	$a_{2}^{\left(1\right)}$	$a_{2}^{\left(2\right)}$	$a_{2}^{\left(3\right)}$	$a_{2}^{\left(4\right)}$
1.8	1.5	0.8	1	0	5	7	10	1.2	1.0	0.9	0.8	0	3	5	10

**Table 2:** AWGN power and Transmission power.

AWGN Power		Transmission Power	
$\sigma_{e}^{2}$	$\sigma_{a}^{2}$	$p_{1}\left(k\right)$	$p_{2}\left(k\right)$
0.1	0.2	1	0.8

**Table 3:** Attack power levels. Dual system (not attacking or attacking one channel).

Not Attack	To Channel 1			To Channel 2		
	$\left(a_{1}^{\left(1\right)},0\right)$	$\left(a_{1}^{\left(2\right)},0\right)$	$\left(a_{1}^{\left(3\right)},0\right)$	$\left(0,a_{2}^{\left(1\right)}\right)$	$\left(0,a_{2}^{\left(2\right)}\right)$	$\left(0,a_{2}^{\left(3\right)}\right)$
(0,0)	(5,0)	(7,0)	(10,0)	(0,3)	(0,5)	(0,10)

**Table 4:** Attack power levels. Dual system (not attacking, attacking one channel or attacking two channels).

Not Attack	Attacking One Channel				Attacking Two Channels	
(0,0)	**To Channel 1**		**To Channel 2**			
	$\left(a_{1}^{\left(1\right)},0\right)$	$\left(a_{1}^{\left(3\right)},0\right)$	$\left(0,a_{2}^{\left(2\right)}\right)$	$\left(0,a_{2}^{\left(3\right)}\right)$	$\left(a_{1}^{\left(1\right)},a_{2}^{\left(2\right)}\right)$	$\left(a_{1}^{\left(2\right)},a_{2}^{\left(1\right)}\right)$
	(5,0)	(10,0)	(0,5)	(0,10)	(5,5)	(7,3)

## Data Availability

Data are contained within the article.

## Abbreviations

The following abbreviations are used in this manuscript:

CPS	Cyber-physical systems
DoS	denial of service
SINR	signal-to-noise ratio
MDP	markov decision process
MMSE	minimum mean-squared error
AWGN	Additive White Gaussian Noise
QAM	quadrature amplitude modulation
AoI	age of information
